# Perioperative utility of amisulpride and dopamine receptor antagonist antiemetics-a narrative review

**DOI:** 10.3389/fphar.2023.1274214

**Published:** 2023-10-31

**Authors:** Murad Elias, Alexa Gombert, Sulaimaan Siddiqui, Sun Yu, Zhaosheng Jin, Sergio Bergese

**Affiliations:** ^1^ Department of Anesthesiology, Stony Brook University Health Sciences Center, Stony Brook, NY, United States; ^2^ Department of Surgery, Stony Brook University Health Sciences Center, Stony Brook, NY, United States

**Keywords:** amisulpride, antiemetics, dopamine receptor antagonist, haloperidol, droperidol, post-operative nausea and vomiting

## Abstract

Despite advances in antiemetics and protocolized postoperative nausea vomiting (PONV) management, it remains one of the most common postoperative adverse events. In patients who developed PONV despite antiemetic prophylaxis, giving a rescue treatment from the same class of medication is known to be of limited efficacy. Given the widespread use of 5-HT3 antagonists as PONV prophylaxis, another class of effective intravenous rescue antiemetic is in dire need, especially when prophylaxis fails, and rescue medication is utilized. Dopamine antagonists were widely used for the treatment of PONV but have fallen out of favor due to some of their side effect profiles. Amisulpride was first designed as an antipsychotic medication but was found to have antiemetic properties. Here we will review the historical perspective on the use of dopamine receptor antagonist antiemetics, as well as the evidence on the efficacy and safety of amisulpride.

## Introduction

Postoperative nausea and vomiting (PONV) is one of the most common adverse events to occur postoperatively, occurring in up to 30% of patients. In high-risk patients, this number can be as high as 70% (Gress et al., 2020). It is second only to postoperative pain in terms of the most common complaints by patients following surgery. Furthermore, it is a significant source of distress and patient dissatisfaction (Eberhart et al., 2002). With a growing trend towards ambulatory and same day surgeries, it is also a major source of delaying discharges from post-anesthesia care units (PACU) (Chatterjee et al., 2011). A single episode of PONV can delay discharge from the PACU by about 25 min (Habib et al., 2006). This can sometimes lead to unanticipated hospital admission and ultimately lead to an overall increase in healthcare costs (Hill et al., 2000). Being able to identify high-risk patients and treat them with the appropriate prophylaxis can greatly improve patient care and satisfaction.

Risk factors for PONV can typically be grouped into three categories: patient factors, type of anesthetic drug, and surgery-related factors. Patient-specific risk factors for PONV in adults are well established in the literature, with the Fourth Consensus Guidelines for the Management of Postoperative Nausea and Vomiting stating that female sex, non-smoking status, young age, and a history of PONV/motion sickness all increase the risk of PONV. Anesthesia-related risk factors include the use of general versus regional anesthesia, postoperative opioid use, and the use of volatile anesthetics and nitrous oxide. Surgery-related factors include the duration of surgery and type of surgery being performed (laparoscopic, intra-abdominal, gynecologic) (Gan et al., 2020).

The Fourth Consensus Guidelines for the Management of Postoperative Nausea and Vomiting by Gan et al. (2020) state the following in terms of guidelines and recommendations for the prevention and management of PONV: 1) Identify Patients’ Risk For PONV, 2) Reduce Baseline Risk For PONV, 3) Administer PONV Prophylaxis Using 2 Interventions in Adults at Risk for PONV, 4) Administer Prophylactic Antiemetic Therapy to Children at Increased Risk for POV/PONV; As in Adults, Use of Combination Therapy Is Most Effective, 5) Provide Antiemetic Treatment to Patients With PONV Who Did Not Receive Prophylaxis or When Prophylaxis Failed, 6) Ensure General Multimodal PONV Prevention and Timely Rescue Treatment Is Implemented in the Clinical Setting, 7) Administer Multimodal Prophylactic Antiemetics in Enhanced Recovery Pathways.

Weibel et al. (2020) conducted a network meta-analysis of antiemetic for PONV prevention, which found significant difference in the efficacy of available therapeutic options. Some monotherapy options such as aprepitant are of equivalent efficacy to the commonly used combination prophylaxis (ondansetron plus dexamethasone); while other antiemetics (such as metoclopramide and domperidone) have comparable efficacy to placebo. Thus, the choice of antiemetic may be just as important as the number of antiemetics that is administered.

Despite these robust guidelines and advances in antiemetics and protocolized PONV management, PONV remains one of the most common adverse postoperative events. In terms of rescue treatment of PONV after failure of prophylaxis, there are very few prospective trials on this topic. Therefore, there is limited evidence to guide clinical management. Moreover, administering rescue antiemetics from the same drug class in patients who have failed prophylaxis has been found to be ineffective, although very commonly practiced.

A 2022 systematic review on the rescue treatment of postoperative nausea and vomiting was conducted by Gan et al. (2022) to summarize the current evidence on this topic. Using the evidence from their review, they created an algorithm for the treatment of PONV in patients with and without prophylaxis. In patients who received no prophylactic antiemetics, 5-HT3 antagonists (ondansetron) remain first-line therapy for established PONV. If ondansetron was used as prophylaxis, it is not beneficial to re-administer ondansetron or another 5-HT3 antagonists unless it was given greater than 6 h prior to the episode of PONV. Although the optimal combination has not yet been established, it appears that combining antiemetics increases efficacy compared to a single agent. Last, if pharmacologic options fail, certain treatments such as acupuncture, acupressure, ginger, and aromatherapy can be considered, although there is a weak level of evidence behind this. They found the following antiemetics to be the most effective: dopamine antagonists (amisulpride 10 mg or droperidol 1.25 mg), 5-HT3 antagonists (ondansetron 4 mg, palonosetron 0.075 mg, granisetron 1 mg, ramosetron 0.3 mg, or tropisetron 0.5 mg), histamine antagonists (diphenhydramine 12.5 mg, dimenhydrinate 25 mg, or promethazine 6.25 mg), and propofol 20 mg bolus.

Before discussing dopamine receptor antagonist antiemetics in this chapter, it is important to briefly review the pathophysiology behind nausea and vomiting. The nucleus tractus solitarius, a region within the brainstem is the site that controls nausea and vomiting. It receives afferent inputs from multiple sources, such as the glossopharyngeal and vagus nerves, vestibular apparatus, cerebellum, and higher cortical centers. All these inputs further interact within the nucleus tractus solitarius as well as the chemoreceptor trigger zone in the floor of the fourth ventricle. The chemoreceptor trigger zone, also known as the area postrema, lies outside the blood-brain barrier and is in direct contact with the cerebrospinal fluid. This allows substances in the blood and cerebrospinal fluid to interact. These areas have been found to contain histamine (H1), serotonin (5-HT3), cholinergic (M1), neurokinin-1, and D2 dopamine receptors (Horn et al., 2014).

Regarding the antiemetics that are used for PONV prophylaxis, there are four major receptor systems involved: cholinergic (muscarinic), dopaminergic (D2), histaminergic (H1), and serotonergic (5-HT3). Neurokinin-1 (NK-1) receptors are also thought to be involved, as NK-1 antagonists such as aprepitant have been used for PONV. These different receptors can be found in areas that are responsible for nausea and vomiting. For example, there are cholinergic receptors in the vestibular nuclei as well as the vomiting centers. The area postrema contains dopamine, serotonin, and opioid receptors. The nucleus tractus solitarius contains μ-opioid receptor, histamine (H1), cholinergic (M1), and neurokinin-1 receptors (Gress et al., 2020). Finally, cannabinoid receptors (CB1) are also found in nucleus tractus solitaries and area postrema.

Since several pathways exist behind PONV, the current consensus guidelines recommend that high risk patients should be given a combination of antiemetics with different mechanisms of action (Weibel et al., 2020; Gan et al., 2022). When choosing an appropriate antiemetic, both the class of drug and the timing of administration should be considered. For example, steroids such as dexamethasone are effective when given prophylactically at the beginning of surgery, whereas 5-HT3 antagonists such as ondansetron are most effective when given 30 min before the end of anesthesia (Gan et al., 2020). This review will focus on dopamine receptor antagonist agents such as amisulpride, haloperidol, and droperidol.

## Dopaminergic secantiemetics

### Haloperidol

Haloperidol is a butyrophenone and typical antipsychotic approved by the Food and Drug Administration (FDA) in 1967 for the treatment of schizophrenia. Haloperidol has high levels of antagonism towards D2 receptors in the central nervous system, leading to its strong antipsychotic effects. At lower doses, haloperidol has also been used as an antiemetic, particularly in palliative care. This effect may be due to antagonism of dopaminergic receptors within the area postrema (Dağ et al., 2019). Within the body, haloperidol is confined to the blood with 90% bound to plasma due to its high intrinsic protein binding capacity. The half-life of haloperidol once administered is around 18 h. Once in the bloodstream, haloperidol undergoes metabolism by the liver through glucuronidation, reduction, and oxidation by cytochrome P450, specifically as a substrate of CYP3A4, prior to excretion in bile (Table 1) (Kudo and Ishizaki, 1999).

**TABLE 1:** Pharmacokinetics and notable side effects of dopamine receptor antagonist antiemetics.

Drug name	Volume of distribution	Metabolism	Half-life	Notable side effects
Haloperidol Kudo and Ishizaki, (1999)	9.5–21.7 L/kg	Hepatic	Oral: 14–37 h	Extrapyramidal Reaction, Parkinsonism, Abdominal Pain, Constipation, Drowsiness, Headache, Sialorrhea
			IM: 20 h	
			IV: 14–26 h	
Droperidol Cisewski et al., (2022)	1.5L/kg	Hepatic	IM: 2–4 h	Extrapyramidal Reaction, Dizziness, Neuroleptic Malignant Syndrome, Cardiac Arrhythmia, Prolonged QT interval, Laryngospasm, Bronchospasm
Amisulpride Rosenzweig et al., (2002)	5.8 L/kg	Undergoes minimal metabolism and its metabolites in plasma are largely undetectable	Oral: 12 h	Infusion Site Pain, Hypokalemia, Increased Serum Prolactin, Hypotension
			IV: 4–5 h	

Several studies have investigated the use of haloperidol as an antiemetic within the dose range of 0.5–4 mg. A 2004 meta-analysis of 15 studies and randomized controlled trials conducted between 1962 and 1988 found that both 1 and 2 mg intramuscular haloperidol were effective at limiting PONV 2–4 h after treatment at similar efficacy and with a similar side effect profile to 5-HT3 receptor antagonists (Büttner et al., 2004).

Haloperidol has been linked to several significant side effects, mostly at high doses. Because haloperidol is not selective for the D2 receptor and blocks other receptors such as cholinergic, noradrenergic, histaminergic receptors, it can cause a multitude of side effects (Gao et al., 2008). Like other typical antipsychotics, haloperidol may cause dopaminergic blockage of the substantia nigra. This can cause extrapyramidal side effects including acute dystonia, Parkinsonism, and tardive dyskinesia. Haloperidol, compared to other drugs in its class, has a high affinity to dopamine receptors, which has been linked to lower side effects overall but higher rates of extrapyramidal symptoms (Gao et al., 2008). Another complication of haloperidol is neuroleptic malignant syndrome (Dixit et al., 2013). Haloperidol also has been demonstrated to prolong the QT interval in a dose-dependent manner which may precipitate torsade de pointes. Torsade de pointes has been reported with intravenous, intramuscular and oral administration of haloperidol (Sharma et al., 1998). However, at lower doses such as the 1–2 mg recommended for PONV prophylaxis, haloperidol has lower toxicity. Buttner et al. reported zero records of cardiac adverse reactions and one case of extrapyramidal symptoms at 4 mg out of 1800 patients. The most significant side effect was increased sedation at 5 mg, with a relative risk of 2.09 (95% confidence interval, 1.73–2.52; number needed to treat, 4.4) (Büttner et al., 2004).

### Droperidol

Droperidol is a butyrophenone and central dopamine antagonist approved by the FDA in 1970 for clinical use as an antiemetic and general anesthesia adjuvant, as well as an antipsychotic agent. It is an analogue of haloperidol with a shorter half-life and rapid sedating effects. Additionally, it possesses significant dopamine receptor antagonistic activity. Although its exact mechanism is unknown, droperidol’s effects come from its high affinity for and selective inhibition of dopamine D2 receptors, resulting in reduced dopaminergic transmission within the four dopaminergic pathways. To a lesser extent, it is also thought to inhibit serotonin (5-HT3), muscarinic, and alpha-2 adrenergic receptors (Table 1) (McKeage et al., 2006).

Droperidol is primarily metabolized by the hepatic CYP3A4 system with inactive metabolites excreted via urine and feces. It has a rapid onset of 3–10 min, with a peak effect occurring at approximately 30 min. The elimination half-life of droperidol is between 2–4 h, however its sedative effects can be observed up to 12 h (Cisewski et al., 2022). The onset of its sedative effects is essentially identical when administered intravenously (IV) versus intramuscularly (IM), which provides clinical advantage when IV access is unobtainable. Droperidol has a high volume of distribution and is highly protein-bound, with up to 90% of it bound to plasma protein (Cisewski et al., 2022). Overall, droperidol is characterized by rapid distribution, extensive protein binding, hepatic metabolism, and a relatively short elimination half-life.

The efficacy of droperidol as an antiemetic for the prevention and treatment of PONV has been investigated across multiple studies. In 1998, a randomized, double-blind, placebo-controlled, multi-site study of 2061 adult surgical outpatients at high risk of PONV was carried out by Fortney et al. to observe the effects of droperidol at doses 0.625–1.25 mg in comparison to 4 mg ondansetron and a placebo as a preventative in PONV. Eligibility criteria were based on ASA physical status I or II, between the ages of 19- and 65-years old with a history of motion sickness or PONV after general anesthesia scheduled for outpatient surgery less than 2 h duration. Study patients were limited to those undergoing procedures with high emetogenic potential such as laparoscopic, genitourinary, lower extremity orthopedics, partial mastectomies, or lumpectomies. Individuals were randomly assigned to one of four treatments: placebo (normal saline), droperidol 0.625 mg, droperidol 1.25 mg, or ondansetron 4 mg. IV administration of the assigned drug was conducted 20 min prior to anesthesia induction. A complete response was defined as no emetic episodes and no requirement for rescue antiemetic medications. At 0–2 h postoperatively, a complete response was observed in 320 of 512 patients (63%) in the 0.625 mg droperidol group and 348 of 505 (69%) in the 1.25 mg droperidol group which was significantly higher compared to 236/510 (46%) in the placebo group (*p* < 0.05). The incidence of complete responses at 0–2 h was similar in the ondansetron and droperidol 0.625 groups (62% and 63%, respectively), but significantly greater in the droperidol 1.25 mg group (69%, *p* < 0.05). In the 0–24 h postoperative period, there was no significant difference in complete response between the ondansetron and droperidol 0.625 or 1.25 mg groups, however all groups remained superior to placebo (Fo et al., 1998).

The proportion of patients without nausea in the first 24 h postoperatively was significantly greater with droperidol 1.25 mg when compared with ondansetron 4 mg or droperidol 0.625 mg (43% vs. 29% or 29%, respectively). Rescue medication was used in 164 of 518 patients (32%) in the 0.625 mg droperidol group, 133 of 510 patients (26%), 174 of 515 patients (34%) in the 4 mg ondansetron group, and 235 of 518 patients (45%) in the placebo group. In regard to adverse event reporting and safety, there was no significant difference in adverse events in the droperidol groups compared to the ondansetron group (Fo et al., 1998). Overall, research suggests that 1–1.25 mg droperidol has comparable efficacy as 4–8 mg ondansetron but is significantly superior to placebo when preventing PONV (Fo et al., 1998).

Domino et al. (1999) compared the efficacy and safety of droperidol, ondansetron, and metoclopramide in preventing PONV in meta-analysis. Droperidol was found to be 34% more effective than metoclopramide in reducing postoperative nausea (pooled OR 0.66, 95% CI 0.48, 0.90; *p* = 0.008). Droperidol was 32% more effective than metoclopramide in reducing postoperative vomiting (pooled OR 0.68, 95% CI 0.54, 0.85; *p* < 0.001). Droperidol was found to be equally effective as metoclopramide in preventing postoperative nausea (Pooled OR 0.99); however, ondansetron was found to be 30% more effective than droperidol in preventing postoperative vomiting (pooled OR 0.70, 95% CI 0.52, 0.94; *p* = 0.018). Furthermore, the study recognized that data was substantially variable across studies that compared efficacy of ondansetron and droperidol (Domino et al., 1999).

When comparing the efficacy of odansetron-droperidol combination therapy versus monotherapy in treating PONV, Matsota et al. (2015) found that combination therapy is superior to monotherapy of either drug alone. 127 patients who underwent laparoscopic cholecystectomy while under general anesthesia were included in this study and assigned to Group D (droperidol only), O (ondansetron only), or D + O (droperidol plus ondansetron). Researchers found that throughout the 24-h study period, 35 patients experienced vomiting in group D, 30 in group O and 11 in group D + O [(D + O vs. D, p < 0.05), (D + O vs. O, p < 0.05)]. Their analysis also revealed that the combination therapy was significantly more effective than monotherapy of agents alone in preventing PONV at 30 min, 3 h and 6 h postoperatively (Matsota et al., 2015).

In a 2022 systematic review of rescue treatment of PONV by Gan et al. (2022), 1–1.25 mg of droperidol demonstrated similar efficacy to 4–8 mg ondansetron in prophylaxis naïve patients. Droperidol was also suggested to be superior to dexamethasone and metoclopramide in these studies, however risks of bias may outweigh these findings.

In 2001, FDA to issue a black box warning due to concerns over the proarrhythmic risks of droperidol. Droperidol was specifically thought to be associated with dose dependent prolonged QTc and torsade de pointes (McKeage et al., 2006). However, various retrospective studies disclosed that there is insufficient evidence to support the FDA’s issued warning against the use of the cost-effective drug. Under the Freedom of Information Act, researchers in the Department of Anesthesiology at Duke University reviewed all individual case reports that led to the issuance of the black box warning on droperidol. They determined only 10 cases in which serious cardiovascular events were reported at appropriate doses of 1.25 mg or less. A review of the case reports revealed multiple confounding factors in each case that show no definitive causation to the adverse cardiac event (Habib and Gan, 2003). Most deaths associated with cardiac arrythmias occurred at doses ranging from 25 to 250 mg (White, 2002). However, clinicians are still weary to reimplement the use of droperidol back into their practice.

As with most typical antipsychotics, extrapyramidal symptoms can be observed as a side effect of droperidol use. These include akathisia, tardive dyskinesia, tremors, and muscle rigidity. Additional side effects associated with the use of droperidol include hypotension, sedation, restlessness, dysphoria, and anxiety (Habib and Gan, 2003).

### Amisulpride

Amisulpride is a selective antagonist of dopamine D2 and D3 receptors. It belongs to the benzamide atypical antipsychotic drug class. It has a much higher affinity for dopamine receptors compared to other receptors, such as serotonin or histamine receptors. This selectivity is thought to be responsible for its relatively lower incidence of side effects compared to other antipsychotic medications such as haloperidol and droperidol. Amisulpride displays linear pharmacokinetics, has a bioavailability of 48%, displays low protein binding (17%), and has an elimination half-life of approximately 12 h. It is predominantly eliminated in the urine as the parent compound (Table 1) (Rosenzweig et al., 2002).

Depending on the dose of amisulpride, it can preferentially block presynaptic D2/D3 receptors versus postsynaptic D2/D3 receptors. Low doses preferentially block presynaptic receptors (enhancing dopaminergic transmission) whereas higher preferentially block postsynaptic receptors (inhibiting dopaminergic hyperactivity) (Rosenzweig et al., 2002). This makes it useful at targeting the negative symptoms of schizophrenia at lower dosages of 50–300 mg/day and the positive symptoms at higher dosages of 400–800 mg/day.

Amisulpride has been used orally for the past 30 years in Europe for psychotic disorders such as schizophrenia. At doses between 50–1,200 mg/day, it has a relatively benign safety profile, even in chronic usage (Rein et al., 2000). The effect of amisulpride on the QT interval and consequent risk of Torsades de pointes appear to be minimal other than at extreme overdoses. At doses up to 300 mg/day, its extrapyramidal side effects did not occur more frequently than placebo (Joy et al., 2011). Recently, an injectable form of the drug (single 5 mg IV dose) was shown to be effective at preventing PONV. It did not have more toxicity than placebo and did not prolong the QT interval enough for it to be clinically relevant, according to a randomized, double-blinded, placebo-controlled, multi-center trial published in 2013 (Kranke et al., 2013).

Since then, there have been several randomized, double-blinded, placebo-controlled trials demonstrating the effectiveness of amisulpride in the prevention of PONV in high risk patients, such as a study published in 2018 by Kranke et al. (2018). In their study, they conducted a randomized, double-blinded, placebo-controlled, international multicenter trial in 1,145 adult surgical patients. These patients had three or four risk factors for PONV, as described in the Fourth Consensus Guidelines (Weibel et al., 2020) (female sex, non-smoking status, young age, and a history of PONV/motion sickness as the main risk factors). Patients were randomized to either receive placebo or 5 mg intravenous amisulpride, at the induction of general anesthesia, in addition to one standard, non-dopaminergic anti-emetic (most commonly ondansetron or dexamethasone). The following was recorded for up to 24 h after wound closure: nausea, retching/vomiting, and the use of rescue medication. The primary endpoint of the study was a complete response, which was described as no emesis or rescue medication use for up to 24 h in the postoperative period.

A complete response was observed in 330 of 572 patients (57.7%) in the amisulpride group and 268 of 575 patients (46.6%) of the control group. This was a difference of 11.1%, with a 95% 5.3–16.8, *p* < 0.001. The incidence of emesis was 13.8% in the amisulpride group versus 20.0% in the control group, *p* = 0.003. Nausea was seen in 50% of the amisulpride group, compared to 58.3% of the control group, *p* = 0.002. Rescue medication was used in 40.9% of the amisulpride group, versus 49.4% of the control group, *p* = 0.002. There were statistically significant differences seen in all the endpoints of the study when comparing the amisulpride to the control group. In terms of adverse events, laboratory and electrocardiogram abnormalities occurred no more frequently in the amisulpride group when compared to the control group. The conclusion of the study was that amisulpride was safe and effective as prophylaxis of PONV when given in combination with an antiemetic from a different class to high-risk adult patients undergoing elective surgeries under general anesthesia with inhalational agents (Kranke et al., 2018).

Several other studies have also concluded the safety and efficacy of amisulpride as not only an agent that can be used for prevention of PONV, but also as a rescue treatment. A systematic review and meta-analysis published by Zhang et al. (2020) in 2020 concluded that intravenous amisulpride was safe and efficacious for the prevention and treatment of PONV compared to placebo.

Habib et al. (2019) conducted a randomized, placebo-controlled phase III clinical trial in 2019 investigating the efficacy of amisulpride as a rescue therapy after failed prophylaxis. The study included over 2,200 surgical patients with moderate to high PONV risks, undergoing open and laparoscopic surgeries. Patients were given standard PONV prophylaxis, with the majority of patients receiving ondansetron or dexamethasone. Patients experiencing PONV within 24 h of surgery were randomized to receive a single dose of 5 or 10 mg intravenous amisulpride or matching placebo. Results showed a higher level of response, measured by incidence of post operative emesis and use of rescue medication within 24 h, in patients given the 10 mg dose of intravenous amisulpride as compared to placebo (41.7% vs. 28.5%; *p* = 0.006), and no significant difference between the group given the 5 mg dose compared to placebo (33.8%; *p* = 0.109). Total number of adverse events were similar between groups. The conclusion of the study was that 10 mg intravenous amisulpride was safe and efficacious for the prevention and treatment of PONV compared to placebo.

A 2019 randomized, double-blinded, placebo-controlled study conducted by Candiotti et al. (2019) investigated amisulpride as a rescue option for patients who received no prior PONV prophylaxis. The study included 1988 men and women aged over 18 years undergoing inpatient and outpatient procedures under inhalational anesthesia, selecting for patients who had low to moderate risks for PONV. Five hundred and sixty patients experienced PONV and were randomized equally to placebo or 5 or 10 mg amisulpride administered intravenously. The primary efficacy end point was complete response, defined as no episodes of emesis or use of rescue medication within 24 h after administration of study medication. Results showed complete response in 31.4% in both the amisulpride 5 and 10 mg groups compared to 21.5% in placebo (*p* = 0.016). The adverse event profile of amisulpride at either dose was similar to placebo.

Notably, the two studies differed in their conclusion regarding 5 mg dose as PONV rescue treatment, with Candiotti et al. (2019) reporting significantly higher efficacy over placebo, while Habib et al. (2019) found no significant difference. There are several possible explanations for the differing results, including the higher baseline PONV risks in Habib’s patient cohort, as well as the PONV prophylaxis they received.

In 2017, two concurrent, randomized, double-blind, placebo-controlled trials were investigated by Gan et al. (2017). The authors found that nausea occurred less often in patients who received amisulpride compared to placebo in at least one of the trials (46.9% vs. 33.8%, *p* = 0.026; 57.6% vs. 46.6%, *p* = 0.070). Furthermore, in terms of safety profile, there were no differences in terms of QT prolongation, extrapyramidal side effects, or sedation in the amisulpride versus placebo arms. Moreover, in one of the two trials, they found that amisulpride was superior to placebo in reducing the incidence of PONV in moderate to high-risk patients. Further studies investigated the side effect in safety profile, such as a 2021 randomized, double-blind, placebo-controlled study of healthy volunteers conducted by Fox et al. (2021), which concluded that a single 10 mg dose of IV amisulpride does not have a clinically significant effect on the QT interval, when given alone or in combination with ondansetron.

There is robust literature supporting the efficacy and safety profile of amisulpride for PONV prophylaxis as well as rescue treatment in adult patients (Rein et al., 2000; Joy et al., 2011; Kranke et al., 2013; Gan et al., 2017; Kranke et al., 2018; Candiotti et al., 2019; Habib et al., 2019; Zhang et al., 2020; Fox et al., 2021). Further studies need to be conducted to evaluate the efficacy and safety profile of amisulpride for PONV in the pediatric population. Currently, there is a need for randomized, double-blinded, placebo-controlled studies in the pediatric population.

### Other dopamine receptor antagonist antiemetics

When discussing the efficacy of antiemetics that antagonize dopamine receptors, it is notable to mention the marginally used therapeutics promethazine, perphenazine, prochlorperazine, and metoclopramide. Promethazine is a phenothiazine derivative with antidopaminergic, antihistamine, and anticholinergic properties. Prochlorperazine is also a phenothiazine derivative with similar properties to that of promethazine. Promethazine and prochlorperazine function as direct antagonists at the mesolimbic dopamine receptors and alpha-adrenergic receptors in the brain. Additionally, promethazine acts as an H1-receptor blocker, exhibiting antihistamine effects (Sharma and Hamelin, 2003; Tan et al., 2010).

Perphenazine, a piperazine phenothiazine derivative, operates through postsynaptic inhibition of dopamine receptors. It exerts central and peripheral nervous system effects by stimulating alpha adrenergic receptors and inhibiting histamine and serotonin receptors. Perphenazine has a substantial first‐pass effect resulting in a bioavailability of only about 40%. Approved as an antipsychotic medication in 1957 in the United States, it has been largely supplanted by atypical antipsychotics due to their more favorable side effect profile (Hartung et al., 2015).

Metoclopramide is another dopamine receptor antagonist which has been used as an antiemetic and prophylactic agent for postoperative nausea and vomiting for over 40 years. While extrapyramidal side effects are rare at typical doses (10 mg or less), higher doses are often required for effective antiemetic action. Consequently, it is not as frequently employed as other agents in preventing postoperative nausea and vomiting (Henzi et al., 1999).

## Conclusion

Appropriately screening patients for PONV risk factors and treating them with the appropriate prophylaxis and rescue treatment if needed is an integral component to providing anesthesia care. Despite advances in antiemetics and protocolized postoperative nausea vomiting (PONV) management, it remains one of the most common postoperative adverse events. In patients with multiple risk factors for PONV, this number has been reported to be as high as 70% (Gress et al., 2020). It is a significant source of distress, patient dissatisfaction, delaying discharges from post-anesthesia care units, increases in healthcare costs, and a cause of unanticipated hospital admission (Hill et al., 2000; Eberhart et al., 2002; Chatterjee et al., 2011; Gan et al., 2020).

Current consensus guidelines support the use of multimodal PONV prophylaxis in patients who are at high risk (one or two risk factors or greater) in attempts to reduce the risk of inadequate prophylaxis. Multimodal therapy should consist of drugs from different classes, while utilizing the minimum effective doses. Patient factors, drug availability, and institutional policy will guide what medications are utilized. In children, it is recommended to use a 5-HT3 receptor antagonist such as ondansetron plus dexamethasone, while also minimizing opioids and volatile anesthetics (Weibel et al., 2020).

Although dopamine receptor antagonist agents such as haloperidol and droperidol demonstrate efficacy in PONV prophylaxis, they have undesirable side effects at higher doses, such as excessive sedation, extrapyramidal symptoms, neuroleptic malignant syndrome, torsades de pointe, hypotension, dysphoria (Sharma et al., 1998; Habib and Gan, 2003; Gao et al., 2008; Dixit et al., 2013). Amisulpride, a selective D2 and D3 receptor antagonist has been extensively studied in its use in PONV prophylaxis and treatment. In several studies it displayed superior efficacy when compared to placebo (Habib et al., 2019), while having minimal side effects (Fox et al., 2021). Amisulpride is a safe and effective agent for PONV prophylaxis and rescue treatment in established PONV in the adult population. In the pediatric population, the literature is sparse and further studies should be conducted to evaluate its safety and efficacy when used for PONV prophylaxis and rescue treatment.
